# Population pharmacokinetics of bevacizumab in cancer patients with external validation

**DOI:** 10.1007/s00280-016-3079-6

**Published:** 2016-06-21

**Authors:** Kelong Han, Thomas Peyret, Mathilde Marchand, Angelica Quartino, Nathalie H. Gosselin, Sandhya Girish, David E. Allison, Jin Jin

**Affiliations:** 1 Clinical Pharmacology, Genentech Inc, 1 DNA Way, South San Francisco, CA 94080 USA; 2 Pharsight Consulting Services, Montreal, QC Canada; 3 Pharsight Consulting Services, Marseille, France; 4 GlaxoSmithKline, 709 Swedeland Rd, King of Prussia, PA 19406 USA

**Keywords:** Population pharmacokinetics, Bevacizumab, Cancer, External validation, Japan, Adult, Asian

## Abstract

**Background:**

Bevacizumab is approved for various cancers. This analysis aimed to comprehensively evaluate bevacizumab pharmacokinetics and the influence of patient variables on bevacizumab pharmacokinetics.

**Methods:**

Rich and sparse bevacizumab serum concentrations were collected from Phase I through IV studies in early and metastatic cancers. Bevacizumab was given intravenously as single agent or in combination with chemotherapy for single- and multiple-dose schedules.

**Results:**

Model-building used 8943 bevacizumab concentrations from 1792 patients with colon/colorectal, non-small cell lung, kidney, pancreatic, breast, prostate and brain cancer. Bevacizumab doses ranged from 1 to 20 mg/kg given once every 1, 2 or 3 weeks. A two-compartment model best described the data. The population estimates of clearance (CL), central volume of distribution (V1) and half-life for a typical 70-kg patient were 9.01 mL/h, 2.88 L and 19.6 days. CL and V1 increased with body weight and were higher in males than females by 14 and 18 %, respectively. CL decreased with increasing albumin and decreasing alkaline phosphatase. The final model was externally validated using 1670 concentrations from 146 Japanese patients that were not used for model-building. Mean prediction errors were −2.1, 3.1 and 1.0 % for concentrations, CL and V1, respectively, confirming adequate predictive performance.

**Conclusions:**

A robust bevacizumab pharmacokinetic model was developed and externally validated, which may be used to simulate bevacizumab exposure to optimize dosing strategies. Asian and non-Asian patients exhibited similar bevacizumab pharmacokinetics. Given the similarity in pharmacokinetics among monoclonal antibodies, this may inform pharmacokinetic studies in different ethnic groups for other therapeutic antibodies.

**Electronic supplementary material:**

The online version of this article (doi:10.1007/s00280-016-3079-6) contains supplementary material, which is available to authorized users.

## Introduction

Bevacizumab (Avastin^®^, Genentech Inc.) is a humanized monoclonal immunoglobulin G (IgG) 1 antibody that specifically binds and neutralizes the biological activity of vascular endothelial growth factor A (VEGF-A), a key isoform of VEGF involved in angiogenesis, and a well-characterized pro-angiogenic factor [1]. Bevacizumab causes inhibition of tumor angiogenesis by blocking VEGF-A from binding to its receptors and leads to tumor growth inhibition. Bevacizumab in combination with standard therapy has received marketing authorization for use in the treatment of various cancers including metastatic colorectal cancer (CRC) [2, 3], non-small cell lung cancer (NSCLC) [4], breast cancer [5], renal cell carcinoma [6], cervical cancer [7] and ovarian cancer [8].

A population pharmacokinetic (PK) model has been previously developed [9]. Bevacizumab PK showed dose linearity within the dose range of 1–20 mg/kg, a slow clearance, a volume of distribution consistent with limited extravascular distribution and a terminal half-life of approximately 20 days. Clearance (CL) and central volume of distribution (V1) increased with body weight and were higher in male patients. CL decreased with increasing albumin and decreasing alkaline phosphatase. There has been no evidence for anti-therapeutic antibodies (ATAs) for bevacizumab in metastatic solid tumors based on the large number of historical clinical studies, and ATA was detected in only 0.6 % of the patients with colon cancer (adjuvant setting) [10].

However, the previous analysis had several limitations. Several important covariates were not evaluated in previous analysis including ethnicity (e.g., Asian vs. non-Asian), indications and baseline VEGF-A. First, bevacizumab has been widely used across ethnic groups (e.g., Asian vs. non-Asian), and supplementary Biologics License Applications have been submitted to health authorities for approval of using bevacizumab for new indications or new combinations based on data from limited ethnic groups while the target population contains much broader ethnic groups. Therefore, it is important to evaluate ethnicity (Asian vs. non-Asian) as a covariate. Second, it has been shown that bevacizumab clearance is 50 % higher and exposure is 50 % lower in gastric cancer as compared to other types of solid tumors [11], making it important to evaluate indication as a covariate. Finally, several studies have shown the predictive value of baseline VEGF-A for bevacizumab treatment effect on progression-free survival and/or overall survival, meaning that only patients with high VEGF-A levels may benefit from bevacizumab treatment, for example in gastric cancer [12] and metastatic breast cancer [13]. Therefore, it is important to evaluate baseline VEGF-A as a covariate. The reason why these important covariates were not evaluated in the previous analysis is likely that these evidences showing the importance of these covariates all appeared after the previous analysis, and therefore, the significance of these covariates may not have been fully realized at that time, and/or the data were unavailable at that time.

Other limitations of the previous analysis include utilization of FO (first-order) instead of FOCE (first-order conditional estimation) algorithm in NONMEM [14], limited number of studies (*n* = 6), patients (*n* = 491) and indications (mainly CRC, NSCLC and breast cancer), etc. Therefore, an updated analysis is warranted.

The objectives of the current analysis were to develop a robust population PK model in adult patients with solid tumors and to evaluate the influence of patient variables on bevacizumab PK, which can be used to simulate bevacizumab exposure to optimize bevacizumab dosing strategies.

## Methods

### Patients

Studies in adult cancer patients included in this analysis are summarized in Table 1. Patients with at least one PK sample were evaluated. Serum bevacizumab concentrations were determined at Genentech, Inc., using an enzyme-linked immunosorbent assay that used recombinant human VEGF for capture and a goat antibody to human IgG conjugated to horseradish peroxidase for detection. The lowest limit of quantification (LLOQ) was 78 ng/mL in serum [9]. Concentrations below the LLOQ were omitted. The clinically relevant covariates tested included those related to demographics, biochemical tests, concomitant medications and pathophysiological factors (Table 2). Values of covariates that follow lognormal distribution were log-transformed. Bevacizumab was given via intravenous infusion of 30–90 min in all patients.

**Table 1:** Summary of studies

Study	Indication	Phase	*N* Patients	*N* Samples
*Model-building population*				
AVF0737g [9]	Solid tumors	I	15	332
AVF0757g [9]	NSCLC	II	60	1083
AVF0761g [9]	Solid tumors	I	12	239
AVF0775g	HRPC	II	15	255
AVF0776g [27]	Breast cancer	II	74	910
AVF0780g [28]	CRC	II	65	1077
AVF2107g [29]	CRC	III	215	607
AVF2119g [30]	Breast cancer	III	35	124
AVF3077s [31]	Colon cancer^a^	IV	679	974
BO17704 [32]	NSCLC	III	138	1064
BO17705 [33, 34]	RCC	III	102	397
BO17706 [35]	Pancreatic cancer	III	80	241
BP20689 [36]	Solid tumors	I	37	712
BO21015 [37]	NSCLC	II	251	856
BO21990 [38]	Glioblastoma	III	14	72
Total			1792	8943
*External validation population*				
JO18157	CRC	I	18	422
JO19901 [39]	Breast cancer	II	69	704
JO19907 [40]	NSCLC	II	59	544
Total			146	1670

*CRC* metastatic colorectal cancer, *HRPC* hormone refractory prostate cancer, *N* number of patients or samples included in this analysis, *NSCLC* non-small cell lung carcinoma, *RCC* renal cell carcinoma

^a^Adjuvant setting

**Table 2:** Summary of patient characteristics

	Model-building data		External validation data	
	*N*	Mean (SD) Median [range]	*N*	Mean (SD) Median [range]
BWT	1792	76.8 (24.2) 74.8 [38.6–195]	146	58.6 (19.0) 57.0 [36.4–95.6]
AGE	1792	58.0 (19.5) 59.0 [20.0–88.0]	146	57.2 (15.9) 40.0 [27.0–52.0]
BSA	1675	1.87 (12.8) 1.86 [1.33–3.07]	146	1.59 (10.4) 1.58 [1.17–2.14]
ALBU	1059	38.5 (13.9) 39.0 [19.0–55.0]	146	40.4 (10.3) 40.0 [27.0–52.0]
TPRO	1055	72.3 (8.7) 72.0 [47.0–101]	146	71.4 (7.0) 71.0 [54.0–85.0]
BALT	1074	31.5 (122.1) 22.0 [3.00–696]	146	23.0 (66.9) 18.5 [7.0–114]
BAST	1072	32.6 (101.6) 23.0 [1.10–516]	146	27.1 (65.7) 21.5 [11.0–158]
BALP	1071	161 (90.1) 109 [2.10–1564]	146	289 (49.1) 254 [118–1020]
BBIL	1069	7.87 (73.4) 6.50 [2.00–93.0]	146	10.0 (37.9) 10.3 [3.42–25.6]
BSCR	1753	78.4 (26.9) 74.0 [26.5–212]	146	56.6 (23.1) 55.7 [33.6–88.4]
CRCL	1753	97.3 (37.2) 91.0 [25.4–359]	146	97.5 (28.3) 95.0 [50.6–195]
Gender	1792	Female: 843 (47 %) Male: 949 (53 %)	146	Female: 103 (70.5 %) Male: 43 (29.5 %)
Race	1113	Caucasian: 929 (51.8 %) Asian: 67 (3.7 %) Black: 49 (2.7 %) Hispanic: 17 (0.9 %) Other: 51 (2.8 %)	146	Asian: 146 (100 %)
Renal function	1753	Normal: 891 (49.7 %) Mild impairment: 666 (37.2 %) Moderate impairment: 196 (10.9 %)	146	Normal: 81 (55.5 %) Mild impairment: 58 (39.7 %) Moderate impairment: 7 (4.8 %)
Concomitant treatment	1792	Single agent: 104 (5.8 %) Chemotherapy: 1586 (88.5 %) Interferon alpha: 102 (5.7 %)	146	Single agent: 18 (12.3 %) Chemotherapy: 128 (87.7 %)

*ALBU* baseline albumin (g/L), *BSA* baseline surface area (m^2^), *BWT* baseline body weight (kg), *N* number of patients with available data, *SD* standard deviation, *TPRO* baseline total protein (g/L)

### Population pharmacokinetic modeling

A population PK model was developed using data from 15 studies: AVF0737g, AVF0757g, AVF0761g, AVF0775g, AVF0776g, AVF0780g, AVF2107g, AVF2119g, AVF3077s, BO17704, BO17705, BO17706, BP20689, BO21015 and BO21990. Nonlinear mixed-effects modeling was performed with NONMEM (version 7.2; ICON Development Solutions, Ellicott City, Maryland, USA) [14] using the FOCE method with interaction, Perl-speaks-NONMEM (version 3.5.3; Uppsala University, Uppsala, Sweden) [15] and R 3.0.3 [16]. Several models with various residual error structures and OMEGA matrices were tested to select the optimal base model. The base model included a power function of body size [e.g., total body weight (BWT)] on all PK parameters:

$$ P_{i} = P_{\text{TV}} \times \left( {\frac{{{\text{BWT}}_{i} }}{70}} \right)^{{\theta_{P} }} $$

where BWT_*i*_ = baseline BWT of patient *i*; *P*_*i*_ = typical PK parameters of patients with BWT_*i*_; *P*_TV_ = typical value of PK parameters for patients with BWT of 70 kg; *θ*_*P*_ = exponent for the PK parameter *P*.

The base model was evaluated using either theoretical (0.75 for clearances and 1 for volumes of distribution) [17] or fitted values of exponents *θ*_*P*_. The quality of fit was evaluated using a standard model discrimination process including statistical criteria [i.e., minimum of objective function value (OFV)], adequate estimation of the parameters (e.g., relative standard error <50 %) and graphical representations of goodness of fit. The final model was established in a stepwise manner by forward addition followed by backward elimination of parameter–covariate relationships with a significance level of *p* < 0.01 and *p* < 0.001, respectively (OFV decrease of 6.63 and 10.83 for one degree of freedom based on Chi-squared distribution, respectively).

The effect of *n* covariates at baseline on PK parameters was coded using a multiplicative model:

$$ \theta_{i} = \theta_{\text{TV}} \times {\text{Effect}}_{1,i} \times \cdots \times {\text{Effect}}_{n,i} $$

where *θ*_*i*_ is the typical value of the parameter for patients with a set of covariates *i*, *θ*_TV_ is the typical value of the PK parameter for patients having the covariate values equal to the median of the covariate for all patients, and Effect_1*,i*_ through Effect_*n,i*_ are multiplicative factors of the effects for covariate 1 through *n*, for the set of covariates *i*. The covariate models for both continuous and categorical covariates were chosen to avoid prediction of negative parameter values.

The multiplicative factor was defined using the power function for continuous covariates:

$$ {\text{Effect}}_{i} = \left( {\frac{{{\text{Cov}}_{i} }}{{{\text{Cov}}_{{{\text{reference}}\;{\text{value}}}} }}} \right)^{{\theta_{\text{eff}} }} $$

and defined as follows for categorical covariates:

- if this categorical covariate is equal to 0, then Effect_*i*_ = 1
- if this categorical covariate is not equal to 0, then Effect_*i*_ = e^*θ*eff^

where Effect_*i*_ is the multiplicative factor of the covariate effect for covariate *i* at baseline, Cov_*i*_ is the covariate value, Cov_reference value_ is the median of the covariate for all patients, and *θ*_eff_ is the exponent of the power function to be estimated. e^*θ*eff^ was used for categorical covariates to force a positive value.

For patients with missing value for a continuous covariate Cov_*i*_, the multiplicative factor of Cov_*i*_ was calculated as [18]:

$$ {\text{Effect}}_{i} = \left( {\frac{{\theta_{{{\text{cov}}\;{\text{missing}}}} }}{{{\text{Cov}}_{{{\text{reference }}\;{\text{value}}}} }}} \right)^{{\theta_{\text{eff}} }} $$

where *θ*_cov missing_ is the value of the covariate that was estimated by fitting to the data from all patients with missing information. During the forward addition step, *θ*_cov missing_ was estimated by fitting to the data for all covariates with missing values. During the backward elimination step, θ_cov missing_ was fixed to the estimates obtained from the forward addition step to stabilize the model. All patients with missing value had the same estimate of *θ*_cov missing_.

### Model evaluation and sensitivity analysis

The population PK models were evaluated using diagnostic plots [19, 20], visual predictive check (VPC) [20, 21], prediction-corrected VPC (pcVPC) [22], bootstrapping [23] and shrinkage [24] assessments. VPC, pcVPC and bootstrapping were all performed using 2000 replicates based on the final model. The relative impact of each covariate included in the final model alone on PK parameters and exposure was explored. Exposure including the trough (*C*_min_) and peak (*C*_max_) concentration was computed at steady state given bevacizumab of 10 mg/kg once every 2 weeks using the final model. The computation was performed using the extreme covariate values (5th and 95th percentiles) and the equations of the final model.

### External validation

After the final model was built, data from three Japanese studies (JO18157, JO19901 and JO19907, Tables 1 and 2) became available and were subsequently used for external validation. Predicted bevacizumab concentrations (*C*_IPRED_) for the validation population were obtained using post hoc Bayesian forecasting by fixing the parameters in the structural and variance models to the final estimates. Prediction errors (PEs) as a measure of bias were calculated for each concentration as PE = (*C*_IPRED_ − *C*_OBS_)/*C*_OBS_, where *C*_OBS_ denotes observed concentrations. Root mean squared prediction error (RMSE) as a measure of precision was calculated as $\sqrt {\frac{1}{n}\sum (C_{\text{IPRED}} - C_{\text{OBS}} )^{2} }$, where *n* denotes the number of observations. pcVPC approach was used to compare the 95 % prediction interval and OBS.

Predicted PK parameters (*P*_IPRED_) for each patient were calculated based on individual covariate values using the equations in the final model without considering observed concentrations. Post hoc estimates of PK parameters (*P*_EST_) were obtained based on observed concentrations and the final model. PE was calculated as (*P*_IPRED_ − *P*_EST_)/*P*_EST_. RMSE was calculated as $\sqrt {\frac{1}{n}\sum (P_{\text{IPRED}} - P_{\text{EST}} )^{2} }$, where *n* denotes the number of patients.

## Results

### Patients

A total of 8943 bevacizumab serum concentrations from 1792 adult cancer patients in 15 studies were included in the model-building dataset, and 1670 concentrations from 146 adult patients in three Japanese studies were included in the external validation dataset. Studies and patient characteristics are summarized in Tables 1 and 2. Less than 5 % of the samples were below LLOQ, and all of them were pre-dose samples.

### Population pharmacokinetic modeling

The optimal base model was a linear two-compartment model with theoretical exponents estimated for clearance (CL), inter-compartment clearance (Q), central (V1) and peripheral (V2) volumes of distribution, full block inter-individual variability (IIV) on CL, V1 and V2 with combined additive and proportional residual error. Parameter estimates of the base model are presented in Supplementary Table 1. In the base model, the estimates of typical bevacizumab CL, V1, Q, V2 and terminal half-life values for a 70-kg patient were 9.01 mL/h, 2880 mL, 18.7 mL/h, 2571 mL and 19.6 days. Thirty-eight covariate relationships were evaluated in the forward addition step. After adjusting for total body weight (BWT), CL and V1 were still higher in male patients. CL decreased with increasing albumin (ALBU) and decreasing baseline alkaline phosphatase (BALP). CL was also lower in patients treated with interferon alpha (Supplementary Fig. 1). No covariate was excluded during the backward elimination step (*p* < 0.001).

Parameter estimates of the final model are summarized in Table 3. Bevacizumab CL and V1 for the patient *i* were described as follows (ALBU = 41.8 and BALP = 76.3 if missing):

$$ {\text{CL}}_{i} = 8.60 \times \left( {\frac{{{\text{BWT}}_{i} }}{70}} \right)^{0.589} \times \,\left( {\frac{{{\text{ALBU}}_{i} }}{39}} \right)^{ - 0.473} \times \,\left( {\frac{{{ \ln }({\text{BALP}}_{i} )}}{\ln (109)}} \right)^{0.312} \times (1.14\;{\text{for}}\;{\text{males}}) \times (0.844\;{\text{for}}\;{\text{interferon}}\;{\text{alpha}}\;{\text{treatment}}) $$

$$ {\text{V}}1_{\text{i}} = 2678 \times \left( {\frac{{{\text{BWT}}_{\text{i}} }}{70}} \right)^{0.470} \times \left( {1.18\;{\text{for}}\;{\text{males}}} \right) $$

**Table 3:** Parameter estimates of the final model in adult cancer patients

Parameter	Estimate	Shrinkage (%)	Bootstrap	
			Median	95 % CI
CL (mL/h)	8.6		8.6	[8.37, 8.82]
V1 (mL)	2678		2678	[2616, 2736]
Q (mL/h)	18.6		18.7	[16.6, 21.3]
V2 (mL)	2423		2417	[2291, 2568]
BWT on CL and Q^a^	0.589		0.586	[0.501, 0.666]
Male on CL^b^	1.14		1.15	[1.11, 1.19]
ALBU on CL^a^	−0.473		−0.474	[− 0.619, −0.323]
Missing ALBU on CL	41.8 g/L			
BALP on CL^a^	0.312		0.321	[0.132, 0.526]
Missing BALP on CL	76.3 U/L			
IFNa on CL^a^	0.844		0.843	[0.780, 0.905]
BWT on V1 and V2^a^	0.470		0.469	[0.396, 0.541]
Male on V1^b^	1.18		1.18	[1.13, 1.22]
Prop. error (%)	21.8	12.0	21.7	[20.7, 22.9]
Add. error (μg/mL)	0.0553	12.0	0.0553	[0.0438, 0.0678]
IIV CL (%)	29.2	17.7	29	[27.2, 31.0]
IIV V1 (%)	18.3	44.3	18.2	[15.6, 20.9]
IIV V2 (%)	41.4	43.8	41.8	[33.2, 49.3]

*Add.* additive, *ALBU* baseline albumin, *BALP* alkaline phosphatase, *CI* confidence interval, *CL* clearance (mL/h), *IIV* inter-individual variability, *IFNa* interferon alpha treatment, *Prop.* proportional, *Q* inter-compartment clearance, *V1* central volume of distribution, *V2* peripheral volume of distribution

^a^Value of the exponent *θ*_eff_ estimated in the model

^b^Values calculated as “e^*θ*eff^,” where *θ*_eff_ is the value of covariate effect for male estimated with the model

### Model evaluation and sensitivity analysis

**Fig. 1:**
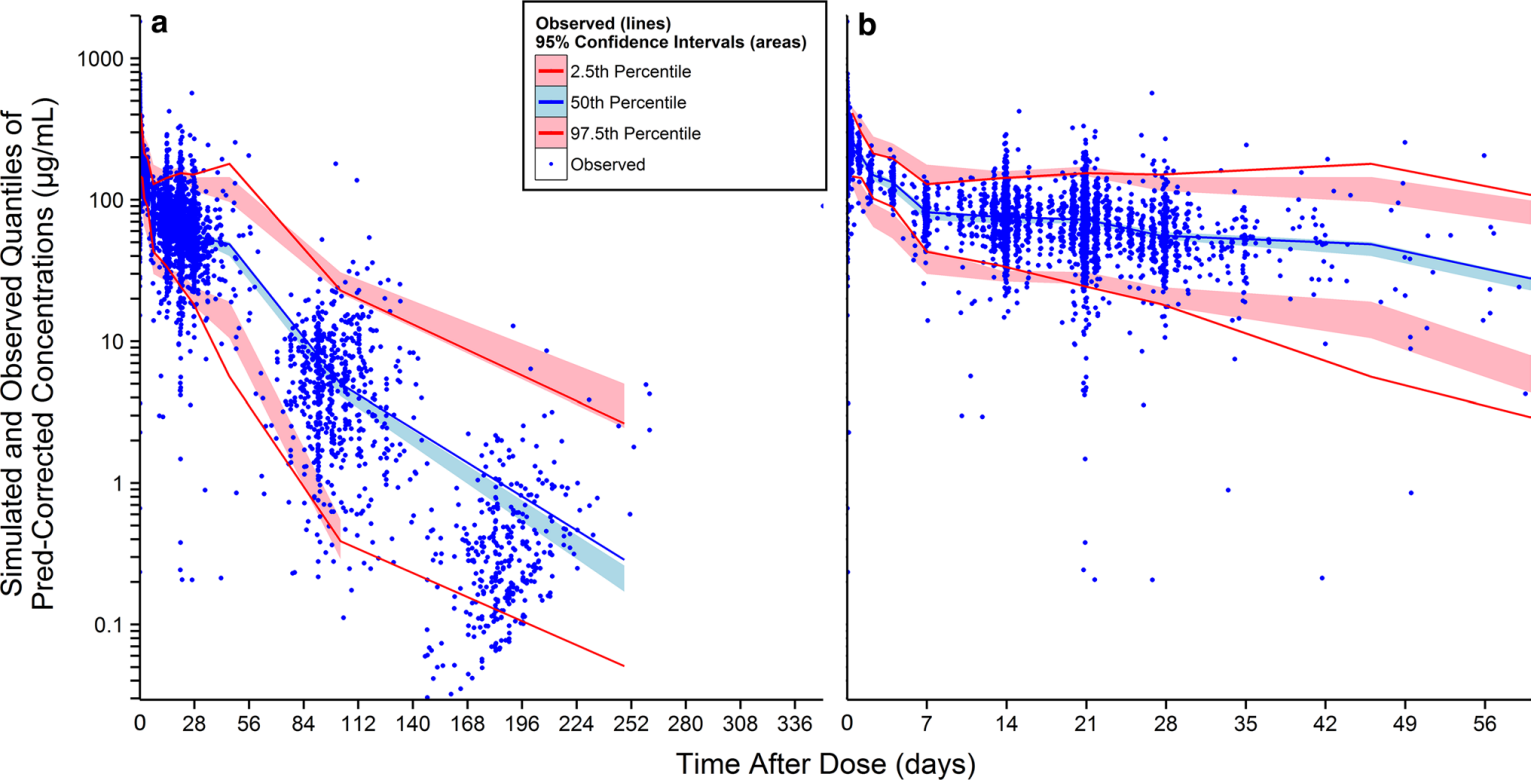
Prediction-corrected visual predictive check for the serum concentration-time profiles of bevacizumab using the final model in adult cancer patients. *Pred* population prediction; figure on the *right* is the part of figure on the *left* during the first 2 months after dose

Goodness-of-fit plots showed good agreement between predicted and observed bevacizumab concentrations with no apparent bias in residual (Supplementary Fig. 2). The pcVPC result for the final model is presented in Fig. 1. Overall, the 2.5th, 50th and 97.5th percentiles of observed concentrations were within the predicted 95 % confidence interval (CI) of these percentiles, suggesting accurate model fitting across a wide range of dosing regimens and time courses. Bootstrapping resulted in median parameter estimates and 95 % CIs similar to the estimates from the original dataset, indicating that the final model provided good precision for parameter estimation.

**Fig. 2:**
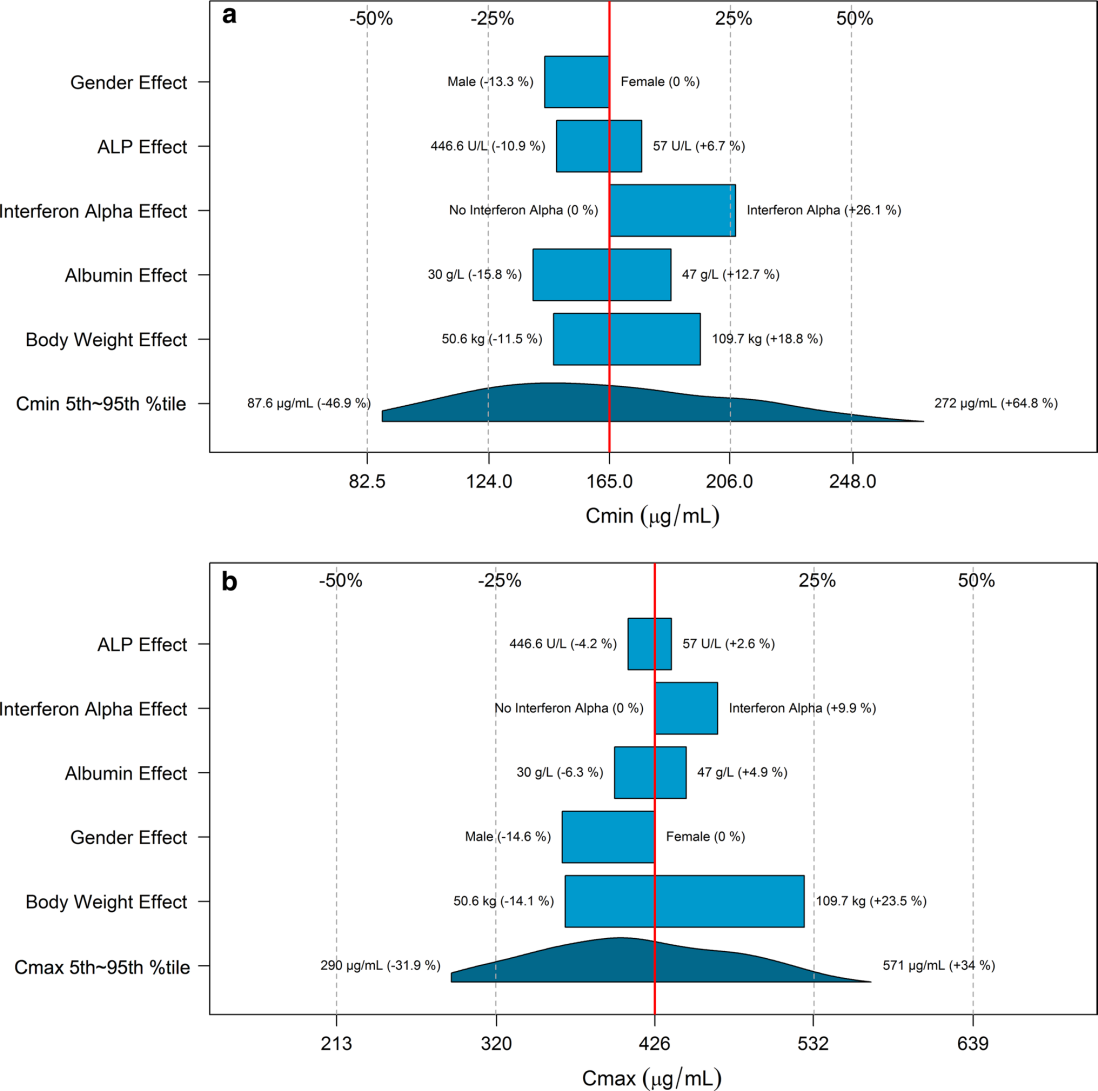

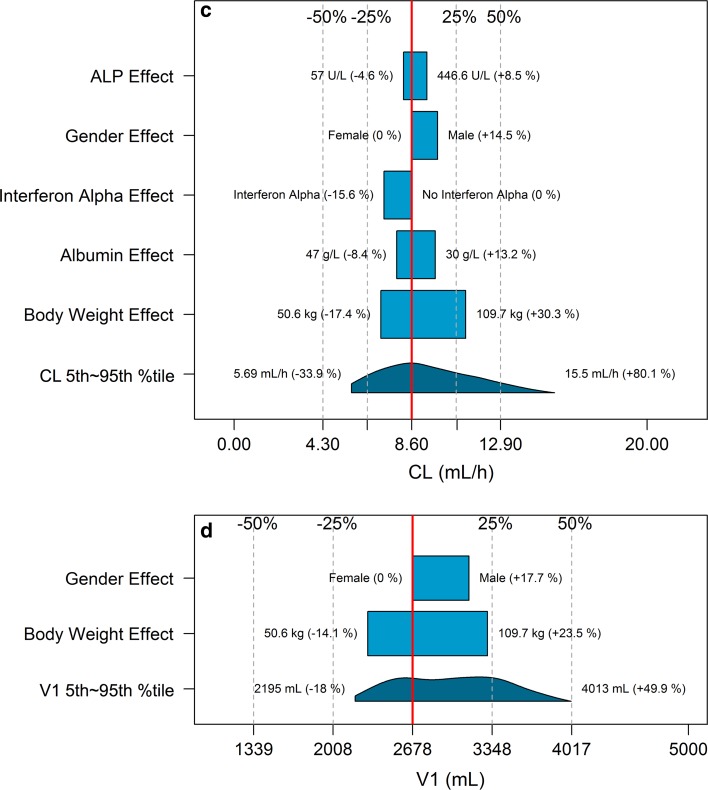
Impact of the variation for a single covariate included in the final model on steady-state bevacizumab exposure and PK parameters in adult cancer patients: **a** *C*_min_ (minimum concentration); **b** *C*_max_ (maximum concentration); **c** CL (clearance); **d** V1 (central volume of distribution). *Red* *vertical lines* represent the “*base*” defined as the exposure or PK parameter estimate of a typical patient, i.e., a 70-kg female patient with albumin of 39 g/L and baseline alkaline phosphatase of 109 U/L without interferon alpha treatment. The *dark blue* *shaded* curve at the *bottom* with value at each end shows the 5th to 95th percentile range of exposure or PK parameter estimate across the entire population. Each *light blue* *shaded* *bar* represents the influence of a single covariate on the steady-state exposure after repeated bevacizumab dose of 10 mg/kg once every 2 weeks or on the PK parameter. The label at *left* end of the bar represents the covariate being evaluated. The *upper* and *lower* values for each covariate capture 90 % of the plausible range in the population. The length of each bar describes the potential impact of that particular covariate on bevacizumab steady-state exposure or PK parameters, with the percentage value in the parentheses at each end representing the percent change from the “*base*.” The most influential covariate is at the *bottom* of the plot for each exposure metric or PK parameter

The impact of the variation for a single covariate included in the final model on steady-state exposure (Fig. 2a, b), CL (Fig. 2c) and V1 (Fig. 2d) and steady-state exposure (Fig. 2) is demonstrated by comparing the simulated CL, V1 and exposure of patients with extreme covariate values (5th and 95th percentiles) to a typical patient with median covariate value. Among all covariates, BWT had the strongest impact on CL (change at extreme BWT values: −17.4 to 30.3 %) and V1 (change at extreme BWT values: −14.1 to 23.5 %). The impact of other covariates on CL (<22 %) and V1 (<18 %) was low. BWT had the strongest impact on *C*_min_ (30.3 %) and *C*_max_ (37.6 %). The impact of the variation for other covariates on *C*_min_ and *C*_max_ was all below 30 %.

### External validation

**Fig. 3:**
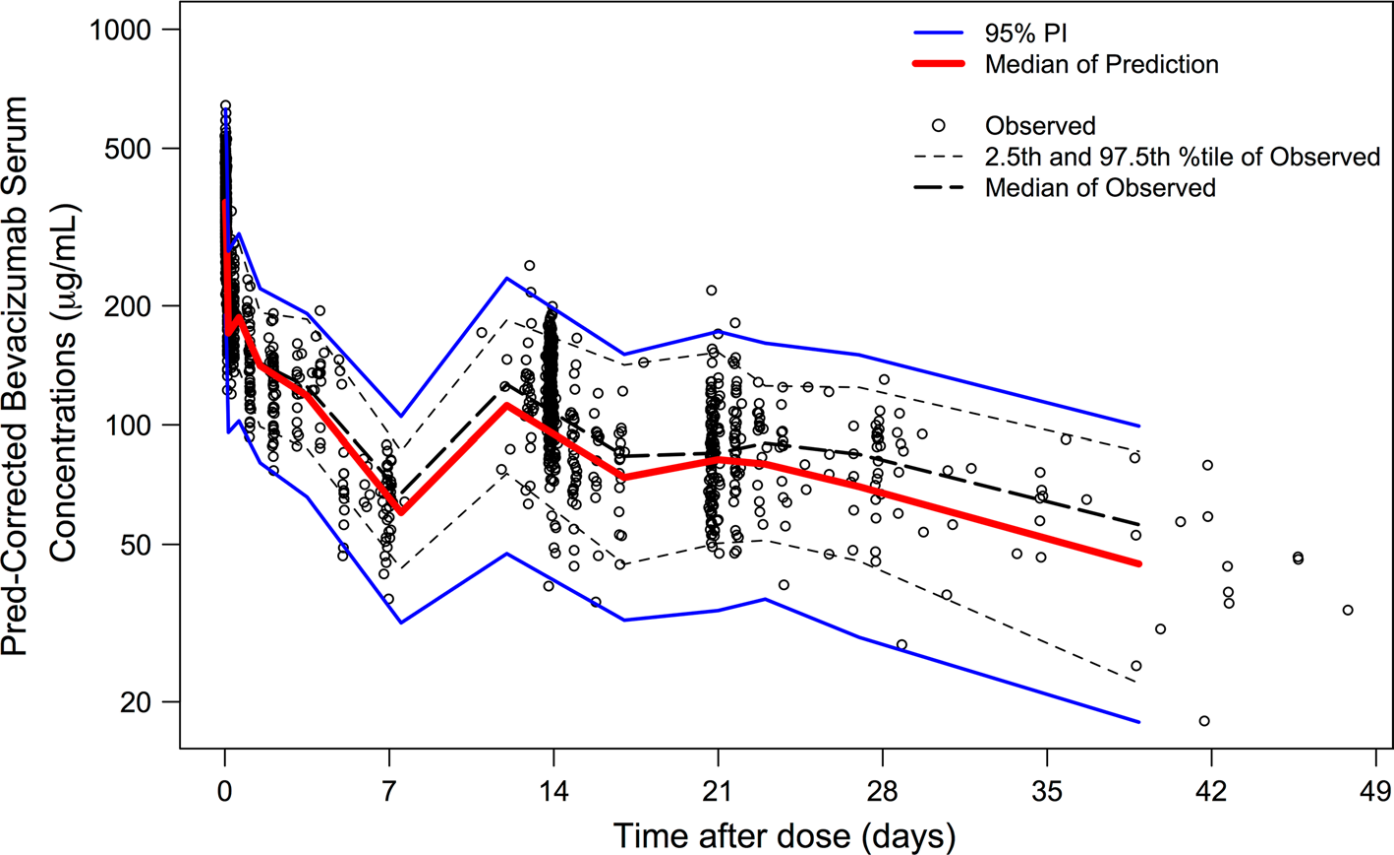
External validation. Most of prediction-corrected observations fall between the 95 % prediction intervals. There is no apparent systematic bias in prediction

**Fig. 4:**
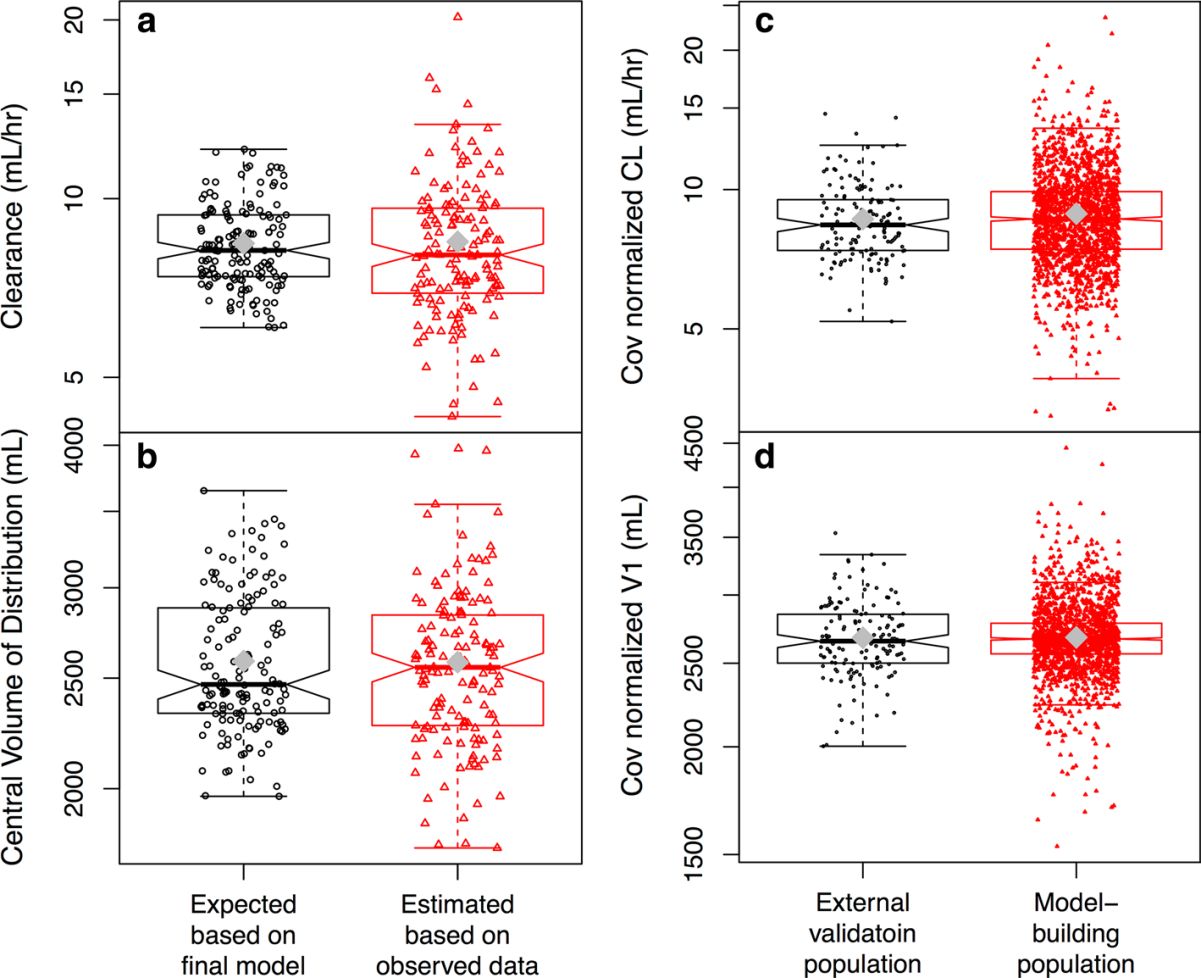
Comparison between (**a**, **b**) individual CL (**a**) and V1 (**b**) calculated based on individual covariate values using the equations in the final model without considering observed concentrations and post hoc estimates of CL and V1 obtained based on observed concentrations and the final model in the external validation population, and between (**c**, **d**) post hoc Bayesian estimates of CL (**c**) and V1 (**d**) of the model-building population and external validation population after normalization by individual covariate values that were included in the final model. *Gray* diamond in the boxplots represents the mean. *CL* clearance, *Cov* covariates included in the final model. *V1* central volume of distribution. In Fig. 4c, d, data points with CL < 3 mL/h (*n* = 2) or V1 < 1500 mL (*n* = 1) are not displayed

Over 95 % of prediction-corrected observations fell within the 95 % prediction interval (PI) (Fig. 3). CL and V1 calculated based on the equations in the final model (*P*_IPRED_) were similar to those estimated based on observed concentrations (*P*_EST_) (Fig. 4a, b). Mean PE for bevacizumab serum concentrations, CL and V1 were −2.1, 3.1 and 1.0 %, respectively. No bias in PE was observed over time and across predicted values. RMSE for bevacizumab serum concentrations, CL and V1 were 0.283, 0.017 and 2.60, respectively.

Post hoc Bayesian estimates of CL and V1 in the model-building population (mostly non-Asian patients) and external validation population (Japanese patients only) were also similar after normalization by individual covariate values that were included in the final model (Fig. 4c, d). The normalization was done by dividing the post hoc Bayesian estimates of CL and V1 by the individual covariate values in the form that appeared in the equations of the final model.

## Discussion

This analysis is a comprehensive PK evaluation of bevacizumab in adult cancer patients in Phases I–IV studies as a single agent or in combination with chemotherapy for both single- and multiple-dose administration with both rich and sparse bevacizumab serum concentration data. A robust population PK model was built based on a large PK population of 1792 patients from 15 studies and then externally validated using data from 146 Japanese patients in three independent studies. This model consolidated all bevacizumab PK data in one model, can timely support simulations and decision making when needed, can help develop consistent pharmacokinetic messages of bevacizumab for investigators and health authorities given that multiple PK models have been developed for bevacizumab and contained inconsistent messages, and can support future studies of bevacizumab in other indications. As mentioned in the “Introduction,” many important covariates that were not evaluated in the previous analysis (e.g., Asian vs. non-Asian, indications, baseline VEGF-A) were evaluated in this analysis.

Typical population PK parameter estimates were similar as previously published [9]. The low IIV of 29 and 18.3 % observed for CL and V1 was typical for antibody drugs [25]. The pcVPC demonstrated adequate fit and predictive performance of the final model (Fig. 1). The median prediction (blue band) may appear to be slightly below the median observation (blue line) beyond day 112, suggesting a possible tendency of under-prediction. However, this tendency is likely irrelevant given (1) the small degree of under-prediction, (2) the sparseness of data beyond day 112, (3) the good predictive performance for 2.5th and 97.5th percentiles (red) across all time points, as well as (4) the complexity and heterogeneity of the data. The external validation (Figs. 3, 4) demonstrated good predictive performance of the final model with no apparent systemic bias and the similarity in bevacizumab PK between Asian and non-Asian adult cancer patients. Although there may appear to be a tendency of over-prediction of the variability (Fig. 3), this tendency is likely irrelevant because the model was built based on more heterogeneous data (15 studies over a decade across various ethnic groups) while the validation data were more homogeneous (three studies over a few years in Asian patients only).

Factors significantly associated with bevacizumab PK were similar as previously published [9]: CL and V1 increased with BWT and were higher in males, and CL decreased with increasing albumin and decreasing BALP. It is well known that CL of other IgG antibodies is faster in patients with lower serum albumin levels [25], likely due to two reasons. First, the level of albumin correlates with disease status. Second, the recycling of albumin and IgG is both mediated by FcRn (neonatal Fc receptor) [25], and therefore, albumin levels may reflect the abundance and efficiency of FcRn. The effect of BALP on bevacizumab CL is likely because BALP is an indicator of disease burden, such as liver or bone metastases. CL was found to be 15.6 % lower in patients treated with interferon alpha. However, this effect was within the overall PK variability and therefore may be clinically irrelevant.

Similar to the previous analysis [9], tumor burden was not included in the final model in this analysis. Among solid tumors, tumor burden is usually an indicator of disease severity and health status. It is usually defined as the sum of longest diameters of target lesions under RECIST (Response Evaluation Criteria in Solid Tumors) criteria for systematic tumors and under other criteria for other tumors (e.g., brain tumors). Inclusion of tumor burden in bevacizumab PK model may not be crucial. First, tumor burden as an indicator of disease burden and health status could already be represented by albumin and BALP in the model. Second, tumor burden as a source of VEGF-A (target of bevacizumab) is irrelevant for bevacizumab PK because bevacizumab molar concentration is thousands of times higher than that of VEGF-A [10], and there has been no evidence of target-mediated drug disposition (TMDD) for bevacizumab [10]. Third, in previous analyses, tumor burden alone showed relatively low impact on bevacizumab exposure in the sensitivity analysis (similar to Fig. 2, data not published). Finally, the final model demonstrated adequate fitting and superior predictive performance without incorporating tumor burden.

On the other hand, three factors made it impossible to test baseline tumor burden as a covariate in this analysis. First, tumor response criteria were inconsistent across these 15 studies that were conducted across a time span of over a decade. Several different versions of RECIST and other criteria (e.g., Macdonald criteria for glioblastoma in BO21990) were used. Second, the methods used to measure tumor burden were inconsistent across studies, such as CT (computerized tomography) scans and MRI (magnetic resonance imaging). Finally, unit of length (mm) and area (mm^2^) both exist in tumor burden data, which cannot be converted to each other. In fact, inclusion of tumor burden in the model would greatly reduce the applicability of the model due to the continuous advancement in tumor response criteria and measurement methods, and due to different tumor response criteria and measurement methods across cancer types, for example RECIST version 1.0 versus version 1.1, RECIST criteria versus Macdonald criteria or RANO (Response Assessment in Neuro-Oncology) criteria, CT scans versus MRI.

In conclusion, a robust population PK model for bevacizumab in adult cancer patients was built and externally validated, which may be used to simulate concentration-time profile in adult cancer patients in future studies [11, 26]. Baseline body weight, albumin, alkaline phosphatase and gender were the covariates with the greatest influence on bevacizumab CL and V1, supporting body weight-based dosing of bevacizumab. No difference in bevacizumab PK was observed between Asian and non-Asian patients. Given the similarity in PK among many monoclonal antibodies, this may inform PK studies in different ethnic groups (e.g., Asian vs. non-Asian) for other therapeutic antibodies without TMDD and significant race-dependent target expression.

## Electronic supplementary material

Below is the link to the electronic supplementary material.

Supplementary material 1 (DOCX 15 kb)
