# Supplementary material for: Population pharmacokinetics of bevacizumab in cancer patients with external validation
Source: Cancer Chemother Pharmacol. 2016 Jun 21;78:341–51. doi: 10.1007/s00280-016-3079-6 (PMC4965493; doi:10.1007/s00280-016-3079-6)
Supplement: Supplementary file 1 — Supplementary material 1 (DOCX 15 kb) [file 280_2016_3079_MOESM1_ESM.docx]

**Supplementary Figure 1.** Correlation between weight-adjusted PK parameters obtained from the bevacizumab base model and patient variables in adult cancer patients. Gray line represents the locally weighted scatterplot smoothing (LOESS); Dash line represents the typical value. ALBU: baseline albumin (g/L); BALP: baseline alkaline phosphatase (U/L); BALT: baseline alanine aminotransferase (U/L); BAST: baseline aspartate aminotransferase (U/L); BBIL= baseline total bilirubin (µmol/L); BSCR: baseline serum creatinine (μmol/L); BWT: baseline body weight (kg); CL.KG: weight-adjusted clearance (mL/hr); LOESS: locally weighted scatterplot smoothing; r: Pearson correlation coefficient; TPRO: baseline total protein (g/L); V1.KG: weight-adjusted central volume of distribution (mL). In the boxplots, black dots and black lines represent the mean and median value in each group, respectively.

**Supplementary Figure 2.** Goodness-of-fit for the final bevacizumab population pharmacokientic model in adult cancer patients. Conc.: concentration; IDENT: identity line; LOESS: locally weighted scatterplot smoothing

**Supplementary Table 1.** Parameter estimates of the base model in adult cancer patients

| **Parameter** | **Estimate** | **IIV** |
| --- | --- | --- |
| CL (mL/hr) | $9.01\times\left( \frac{\mathrm{BWT}}{70} \right)^{0.712}$ | 32.7 |
| V1 (mL) | $2880\times\left( \frac{\mathrm{BWT}}{70} \right)^{0.585}$ | 20.1 |
| Q (mL/hr) | $18.7\times\left( \frac{\mathrm{BWT}}{70} \right)^{0.712}$ | NA |
| V2 (mL) | $2571\times\left( \frac{\mathrm{BWT}}{70} \right)^{0.585}$ | 44.2 |
| Prop (%) | 21.7 |  |
| Add (µg/mL) | 0.0564 |  |

Add: additional residual error; BWT: baseline body weight (kg); CL: clearance; IIV: inter-individual variability (%); NA: not applicable; Prop: proportional residual error; Q: peripheral clearance; V1: central volume of distribution; V2: peripheral volume of distribution
